# Progress in Surgery of the Intestines

**Published:** 1904-01-23

**Authors:** 


### Progress in Surgery of the Intestines.

**Intestinal Anastomosis.**—Bishop[1] formulates the following :—1. It is suggested that the terms "fundal point" and "fundal line" will be useful for purposes of description as defining that portion of the intestine which is opposite to the mesenteric insertion. 2. The use of decalcified bone bobbins is advisable in operations for union in the gastric region and the upper part of the small intestine, mainly because they act as a protection during the early stages of union, and because the intestinal contents in these segments are liquid, and possess certain chemical and digestive properties, from which the line of union in these regions needs to be protected. 3. The use of such appliances is doubtful in the lower small intestine, and is absolutely contraindicated in the large, mainly because of the solid or semi solid nature of their contents. This objection does not apply to Wackerhagen's wafers, which indeed are to be restricted to these segments of the gut. 4. In choosing a mode of suture, that one is to be preferred which gives the most perfect security against leakage in consequence of its yielding to the peristaltic strain, exposes itself the least, and so minimises the irritation and consequent adhesion of surrounding peritoneal surfaces, by which later obstruction may be produced, and which removes the inevitable diaphragm formed by the necessary inturning of the bowel ends, so as to leave a smooth internal surface. 5. The use of forceps, such as O'Hara's, or the preliminary crushing of the intestine, as in Vidal's or Doyen's methods, all of which are based upon the idea of producing sphacelation of the inturned edges of the gut, are of very doubtful value, are associated with unnecessary risk of later perforation, and should not be preferred to simple suturing or to suture over some absorbable and protective appliance. 6. In cases where a neoplasm or other obstruction has to be removed from the large intestine, the prospects of success are greater if a colotomy above the seat of the obstruction can be previously carried out. Makins[2] discusses the three methods of joining intestine—the axial or end to end, lateral anastomosis and lateral implantation. Axial anastomosis should be limited to cases where the bowel is invested with a complete peritoneal covering and provided with a free mesentery, so that the intestine can be moved sufficiently to allow the anastomosis to be carried out, while lateral anastomosis is applicable everywhere except at the junction of the sigmoid flexure and rectum. In favour of the axial method, it may be said that when it heals thoroughly the result is very good, and only one joint has to be made, but against it, it may be said that the situation of the junction is not good, for the vessels have to be divided where they are large, and thus the union may not be so firm and the risk of leakage is greater. In favour of lateral anastomosis it might be pointed out that this is the "natural" way in which anastomosis occurs in disease, as in "vicarious communications" between two portions of bowel. The advantages of lateral anastomosis are :—1. It approximates two plain surfaces of intestine instead of corrugated surfaces as in end-to-end union. (2) The size of the opening can be made as large as is necessary, so that there is no risk of cicatricial contraction. (3) It is possible to avoid completely the mesenteric border and thus the intestinal vessels are only divided where they are small. (4) It is possible to avoid the field of disease without removing too large a portion of bowel, as in necrosis of the bowel after strangulation. Moreover, when leakage does occur after lateral anastomosis it is almost always at the blind end of the bowel. Lateral implantation is chiefly applicable to cases where it is desired to unite a piece of bowel of small lumen to one much larger, as in union of the ileum to the ascending colon.

**Volvulus.**—Laparotomy should be performed without delay. Moynihan[3] says if a volvulus be found it is brought to the surface and its condition examined. If readily untwisted and the gut in fair condition, the bowel may be returned forthwith. If, however, the case is of long standing, an enormous distension of the coil may be found, and unravelling may be impossible until the bowel has been incised and its contents evacuated. This is the desirable course in many, if not in all instances, for the emptying of the gut allows of its easy reposition, lessens the likelihood of septic absorption from the distension ulcers of the gut, and permits an early restoration of the peristaltic movements of the intestine. In a few cases the condition of the involved loop is of doubtful vitality. If so, a Paul's tube may be inserted into the gut, or the loop may be placed immediately beneath the abdominal wound and a drainage tube or ample gauze packing passed down to the gut. If the involved loop be actually in a state of gangrene, or if the volvulus prove to be due to a mesenteric growth which is perhaps malignant in nature, or if there be many adhesions to the loop, and the bowel become lacerated in separating them, an excision of the affected gut should be performed. After the untwisting of a volvulus there may be a marked tendency to recoiling, the bowel may seem to spring back at once into its former twisted position, but this is lessened by the emptying of the gut. In such cases the loop should be stitched to the anterior abdominal wall, or in the case of the sigmoid ends of the loop should be sutured to the iliac fossa and the side of the pelvis. In all instances of chronic or recurring volvulus it is probable that excision of the implicated loop will prove the most satisfactory treatment. Senn has treated cases by shortening the mesentery by folding it upon itself in a direction parallel to the bowel. Vaughan[4] deals with volvulus of the small intestine, and records 61 cases extracted from the literature.

Intestinal Perforation in Typhoid Fever is considered by Briggs.[5] The incision in the right lower quadrant of the abdomen is the only logical opening in these cases, and the linea semilunaris opposite or a little above the anterior superior spine he believes to be preferable. After entering the abdomen the first search for the perforation should be made in the ileum immediately above the cæcum, as the vast majority of perforations occur in this portion of the gut. Irrigation of the abdominal cavity, he thinks, should be somewhat prolonged and thorough, even if there are no visible evidences of peritonitis or extravasation. These patients bear operation much better than is usually supposed. In instances where no perforation is found, either with or without evidences of peritoneal infection, at once arises the question of how far one may feel justified in exploring the abdomen for such other intra-abdominal conditions as have been known to suggest perforation. Where, however, one finds an abnormal intra-abdominal condition, as peritonitis, for instance, it seems hardly justifiable to limit the search for the seat of the trouble to a rapid glance at the ileum and cæcum. The appendix is so close at hand that its inspection should never be omitted. The detection of suppurating mesenteric glands is important for the sake of adequate disposition of drainage. Exploration of the gall-bladder may be accomplished without serious objection and the presence of intestinal obstruction may possibly be detected by a hurried examination of the intestine from several parts of the abdomen. Armour[6] says the majority of surgeons would now appear to advocate early operation. While resection of the ulcerated portion of the bowel with immediate end-to-end anastomosis would be the ideal operation, it can scarcely ever be feasible. The suturing should be done without paring the edges, and all of the thinned area should be included in the suture, care being taken, however, to avoid inverting too much, lest too great a narrowing of the lumen take place.

Intestinal Obstruction.—English[7] advocates the use of saline irrigation in abdominal operations. The following advantages are claimed for this method:—(1) Distension of the intestines is usually greatly diminished and this is a factor of utmost importance, both in regard to the direct effect upon the patient and the care with which the necessary manipulations are carried out. (2) Shock, which always attends these operations, is diminished by the free absorption of saline fluid. (3) Thirst, which is one of the most distressing features after abdominal operations, is undoubtedly diminished as a result of the free absorption of fluid. (4) The influence of irrigation upon the subsequent formation of adhesions is an important point; and where there is a likelihood of peritoneal adhesions following, the best way of minimising or preventing them is to leave as much fluid as possible in the peritoneal cavity. (5) Renal secretion is greatly increased and thus many of the toxic products circulating in the blood are eliminated through the kidneys. The importance of systematically washing out the stomach before operation in cases of intestinal obstruction can scarcely be over-estimated, especially if there be severe stercoraceous vomiting. Interesting cases are recorded by Kempe[8] and Newbolt.[9]

[1] Brit. Med. Jour., Oct. 10, 1903, p. 886. [2] Lancet, Aug. 1, 1903, p. 349. [3] Med. Chron., Feb. 1903, p. 305. [4] Amer. Jour. Med. Sci., May 1903, p. 799. [5] Ibid, May 1903, p. 821. [6] Lancet, Oct. 3, 1903, p. 937. [7] Ibid, Aug. 15, 1903, p. 452. [8] Ibid, Aug. 15, 1903, p. 452. [9] Ibid, Oct. 17, 1903, p. 1092.

